# Generation of novel affibody molecules targeting the EBV LMP2A N-terminal domain with inhibiting effects on the proliferation of nasopharyngeal carcinoma cells

**DOI:** 10.1038/s41419-020-2410-7

**Published:** 2020-04-01

**Authors:** Jinshun Zhu, Saidu Kamara, Danwei Cen, Wanlin Tang, Meiping Gu, Xingyuan Ci, Jun Chen, Lude Wang, Shanli Zhu, Pengfei Jiang, Shao Chen, Xiangyang Xue, Lifang Zhang

**Affiliations:** Institute of Molecular Virology and Immunology, Department of Microbiology and Immunology, School of Basic Medical Sciences, Wenzhou Medical University, 325035 Zhejiang, Wenzhou China

**Keywords:** Tumour virus infections, Tumour virus infections, Diagnostic markers, Diagnostic markers

## Abstract

Nasopharyngeal carcinoma (NPC) induced by latent infection with Epstein-Barr virus (EBV) remains the most common head and neck cancer in Southeast Asia, especially in the southern part of China. It is well known that persistent expression of two EBV latent membrane proteins (LMP1/LMP2A) plays a key role in nasopharyngeal carcinogenesis. Therefore, the therapeutic approach of targeting the LMP1/LMP2A protein and subsequently blocking the LMP1/LMP2A-mediated signalling pathway has been considered for treating patients with NPC. Recently, affibody molecules, a new class of small (~6.5 kDa) affinity proteins, have been confirmed to be powerful generalisable tools for developing imaging or therapeutic agents by targeting specific molecules. In this study, three EBV LMP2A N-terminal domain-binding affibody molecules (Z_LMP2A-N_85, Z_LMP2A-N_110 and Z_LMP2A-N_252) were identified by screening a phage-displayed peptide library, and their high affinity and specificity for the EBV LMP2A N-terminal domain were confirmed by surface plasmon resonance (SPR), indirect immunofluorescence, co-immunoprecipitation and near-infrared small animal fluorescence imaging in vitro and in vivo. Moreover, affibody molecules targeting the EBV LMP2A N-terminal domain significantly reduced the viability of the EBV-positive cell lines C666-1, CNE-2Z and B95-8. Further investigations showed that affibody Z_LMP2A-N_110 could inhibit the phosphorylation of AKT, GSK-3β and β-catenin signalling proteins, leading to suppression of β-catenin nuclear translocation and subsequent inhibition of c-Myc oncogene expression, which may be responsible for the reduced viability of NPC-derived cell lines. In conclusion, our findings provide a strong evidence that three novel EBV LMP2A N-terminal domain-binding affibody molecules have great potential for utilisation and development as agents for both molecular imaging and targeted therapy of EBV-related NPC.

## Introduction

Epstein-Barr virus (EBV), also known as human gammaherpesvirus (HHV-4), was discovered by Epstein and Barr in tumour cell cultures derived from endemic Burkitt lymphoma (BL) patients in 1964^1^. This virus is exceptionally prevalent in humans, and its specific serum antibody can be detected in more than 95% of adults worldwide^2,3^. Several human cancers, such as BL, nasopharyngeal carcinoma (NPC), Hodgkin’s lymphoma (HL) and gastric cancer, have been demonstrated to be linked to EBV infection^4–6^. EBV-related NPC is a malignancy with distinct geographical distribution and has a relatively high prevalence in Southeast Asia, especially in the southern part of China^7,8^. Both radiotherapy and chemotherapy are commonly used in the treatment of NPC patients; however, the prognosis remains poor due to local recurrence and distant metastasis^9^. Diagnostically, NPC symptoms are non-specific at the early stage; thus, approximately 80% of NPC patients progress to the middle-tumour or late-tumour, node, metastasis (TNM) stages^10,11^. Therefore, the development of diagnostic and treatment methods, such as molecular imaging probes and targeting agents, is urgently needed to improve the early diagnosis and clinical outcome of NPC patients.

In NPC, EBV latent gene expression is restricted to latent membrane proteins (LMP1, LMP2A and LMP2B), EBV nuclear antigen (EBNA1), EBV-encoded small RNAs (EBERs), and EBV-encoded BARF1 protein and BART microRNAs^3,12^. Latent membrane proteins are able to hijack critical signalling pathways, including PI3-K/Akt, Wnt/β-catenin, and JAK/STAT, to promote EBV-related pathogenesis and further tumourigenesis^3,12,13^. LMP1 is essential for malignant transformation of B lymphocytes and contributes to the initiation and promotion of NPC^3^. In contrast to LMP2A, LMP2B lacks 119 amino acids of the LMP2A N-terminal cytoplasmic domain and can modulate LMP2A activity^3,12^. LMP2A exerts profound effects on the maintenance of latent EBV infection, affecting a variety of cell processes, including proliferation, survival, invasion and motility^3,12,13^. The 119 amino acids of the LMP2A N-terminal cytoplasmic domain (LMP2A-NCD) include two proline-rich motifs (PPPPY), a YEEA motif and an immunoreceptor tyrosine-based activation motif (ITAM). These motifs contain eight tyrosine kinase domains that are responsible for activation of cellular pathways, leading to expression of genes associated with cell cycle progression, such as c-Myc, cyclin D1, and matrix metalloproteinase 7 (MMP7)^3,14,15^. LMP2A is easily detected in EBV-related NPC patients, with 98% of tumour samples showing RNA transcription levels and 60% expressing protein^3,12,16^. Both siRNA-targeted LMP2A and small molecular inhibitors against LMP2A-related signalling pathways can inhibit the proliferation of NPC cell lines in vitro and in vivo^17,18^. Notably, the PI3K/mTOR dual inhibitor BEZ235 has been used in clinical trials and may be included in the therapeutic programme for EBV-related tumours^19^. Therefore, LMP2A has been identified as an important diagnostic biomarker and a promising target for EBV-related tumour therapy.

Affibody molecules are a recently developed class of small robust scaffold proteins derived from the immunoglobulin G (Ig G) binding domain of *Staphylococcus aureus* protein A (SPA). Thirteen specific amino acids in the three α-helix regions of the IgG binding domain can be randomly mutated to construct an affibody library. Theoretically, this library can be screened to obtain affibody molecules with high affinity and specificity to any given target molecule^20,21^. The binding features of affibody molecules to target molecules are similar to those of antibodies but have some unique advantages over antibodies, such as (i) low immunogenicity, (ii) rapid tumour accumulation and clearance from the blood and non-specific sites, (iii) stable physical and chemical properties, and (iv) easy-to-label molecules (i.e., fluorescein and biotin)^20,21^. To date, more than 500 papers have been published on this topic (www.ncbi.nlm.nih.gov). As high-affinity ligands, affibody molecules specifically target more than 40 membrane molecules or viral oncoproteins, including human epidermal growth factor receptor 2 (HER2)^22^, epidermal growth factor receptor (EGFR)^23^, HIV-1 envelope glycoprotein gp120 (HIV-1-gp120)^24^, and human papillomavirus type 16 E7 (HPV16E7)^25^, showing great potential for in vivo molecular imaging, receptor signal blocking and biotechnology applications^20,21^.

In this study, we describe the generation and characterisation of three novel LMP2A N-terminal domain-binding affibody molecules (Z_LMP2A-N_ affibodies) for their ability to bind to recombinant and native LMP2A-NCD protein and their application to in vivo molecular imaging in tumour-bearing nude mice. Moreover, our data further confirm that Z_LMP2A-N_110, by inhibiting phosphorylation of AKT, GSK-3β and β-catenin signalling proteins, can suppress nuclear translocation of β-catenin, which in turn decreases the expression of c-Myc oncogene and thereby reduces viability of NPC-derived cell lines. To our knowledge, this study is the first report on Z_LMP2A-N_ affibodies as potential agents for molecular imaging and targeted therapy for EBV-related NPC.

## Results

### Selection and expression of Z_LMP2A-N_ affibodies

**Fig. 1:**
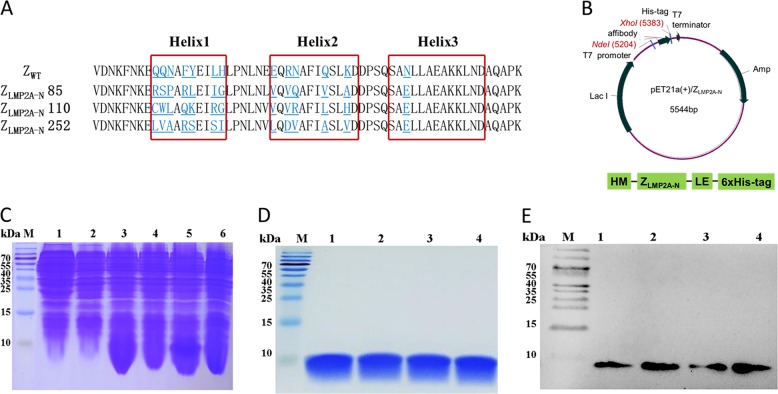
Expression and purification of Z_LMP2A-N_ affibodies. **a** Amino acid sequence alignment of Z_WT_ affibody and Z_LMP2A-N_ affibodies. Thirteen randomised amino acid residues in Z_LMP2A-N_ affibodies are underlined. Red boxes indicate three α-helical subdomains in the wild-type Z domain. **b** Schematic structure of pET21a(+)/affibody recombinant plasmid. **c** Coomassie Brilliant Blue staining SDS-PAGE gel of the recombinant proteins. M, protein ladder; 1, Empty *E. coli* BL21(DE3); 2, *E. coli* BL21 (DE3) transformed with pET21a(+) empty vector; 3–6, *E. coli* BL21(DE3) transformed with pET21a(+)/Z_LMP2A-N_85, pET21a(+)/Z_LMP2A-N_110, pET21a(+)/Z_LMP2A-N_252 and pET21a(+)/Z_WT_ plasmid induced by 1 mM IPTG for 6 h, respectively. The purified Z_LMP2A-N_ affibodies were analysed by SDS-PAGE (**d**) and confirmed by Western blotting (**e**) M, protein marker; 1, Z_LMP2A-N_85; 2, Z_LMP2A-N_110; 3, Z_LMP2A-N_252; 4, Z_WT_.

A total of 65 clones that showed increased interaction with LMP2A-NCD in ELISA experiments (Supplementary Fig. 1B) were selected for DNA sequencing after three rounds of screening of a bacteriophage display library. Sequences were analysed using DNA Star software and further aligned with the sequence of affibody Z_WT_. A total of 59 clones (59/65 or 90.8%) with correct sequences were obtained. Three potential affibodies, Z_LMP2A-N_85, Z_LMP2A-N_110 and Z_LMP2A-N_252, which showed relatively high-yield expression and purification as recombinant proteins in *E. coli* BL21 and high binding affinity in the ELISA screening, were selected for sequence homology analysis. The three affibodies had high homology in the framework region of the affibody but were highly diverse in the helical regions (Fig. 1a).

DNA sequences encoding the three affibody genes were cloned into pET-21a(+) to generate the recombinant plasmid pET21a(+)-affibody (Fig. 1b). After induction with 1 mM IPTG for 6 h at 37 °C (Fig. 1c), His-tag fusion affibodies were purified by affinity chromatography using Ni-NTA agarose resin. SDS-PAGE analysis showed that the purity of the final products was approximately 95% (Fig. 1d), meaning that these products could be used for subsequent investigations. In addition, Western blotting results showed that the fusion proteins could specifically react with anti-His-tag mouse mAbs (Fig. 1e).

### Z_LMP2A-N_ affibodies interacted with LMP2A-NCD with high binding affinity

**Fig. 2:**
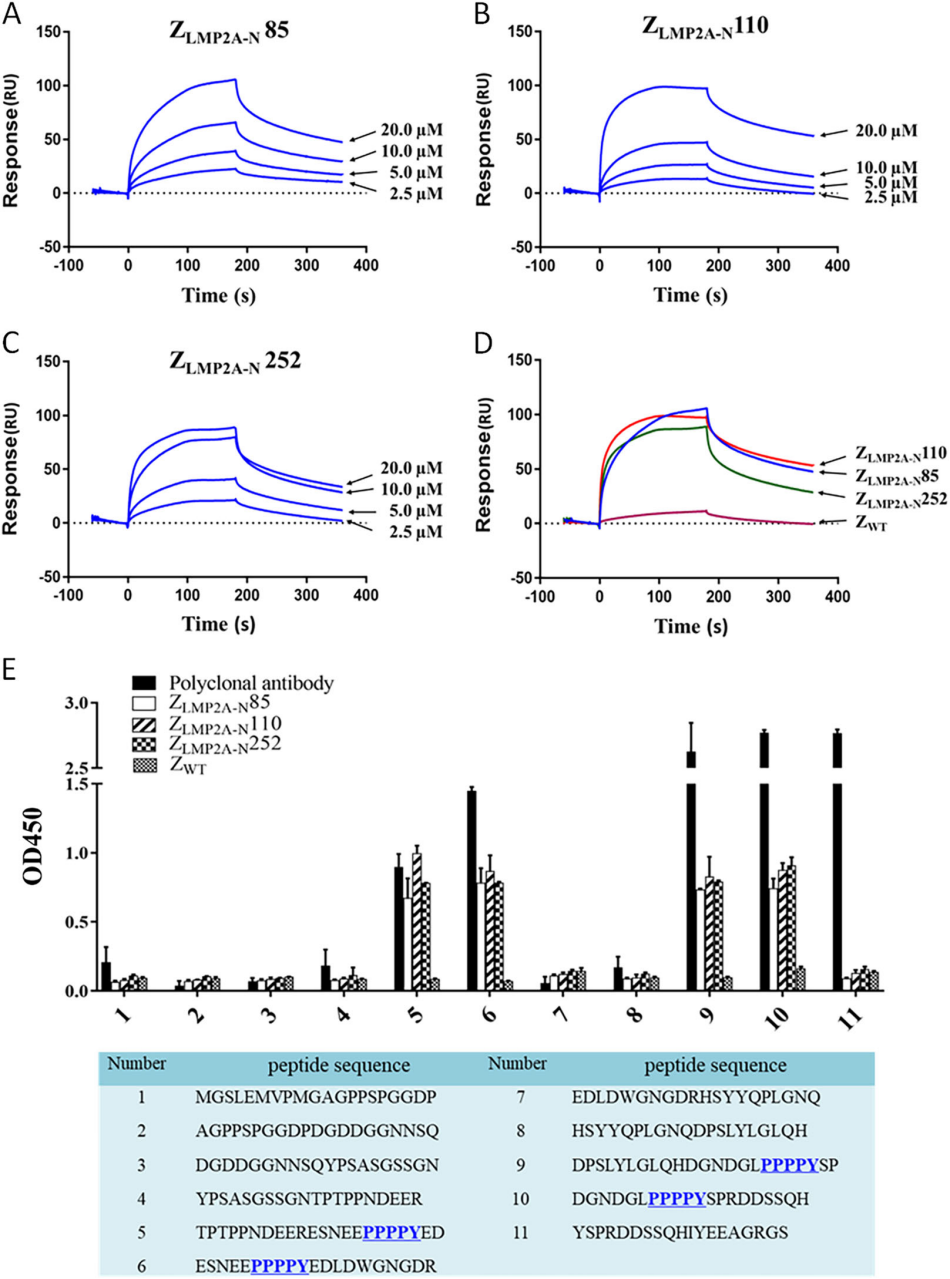
Analysis of the binding properties of the Z_LMP2A-N_ affibodies to recombinant LMP2A-NCD. The binding abilities of different concentrations of purified Z_LMP2A-N_85 (**a**) Z_LMP2A-N_110 (**b**) and Z_LMP2A-N_252 (**c**) to recombinant LMP2A-NCD were tested using a SPR-based binding assay. **d** Sensorgrams obtained after injection of the Z_LMP2A-N_ affibodies at a concentration of 20 μM over a sensor chip containing purified recombinant LMP2A-NCD. The Z_WT_ affibody was used as a control. **e** Epitope mapping of the Z_LMP2A-N_ affibodies by ELISA. The eleven truncated peptides (1–11) listed above were synthesised based on LMP2A-NCD (amino acid, 1–119) and reacted with Z_LMP2A-N_ affibodies and an anti-LMP2A-NCD polyclonal antibody. The underlined sequences highlighted in blue indicated the potential domain recognised by the Z_LMP2A-N_ affibodies. Error bars indicate standard deviations.

A surface plasmon resonance (SPR) biosensor assay was employed to verify the binding ability of Z_LMP2A-N_ affibodies to recombinant LMP2A-NCD using a BIAcore T200 biosensor instrument. Affibody molecules were injected at different concentrations over the chip containing immobilised recombinant LMP2A-NCD. The results showed concentration-dependent increases in resonance signals (Fig. 2a–c) and indicated that all three affibodies could well bind to recombinant LMP2A-NCD. In contrast, Z_WT_ affibody could not be detected in any effective reaction units in resonance signals (Fig. 2d). Moreover, kinetic BIAcore analysis showed that the dissociation equilibrium constants (KD) of Z_LMP2A-N_85, Z_LMP2A-N_110 and Z_LMP2A-N_252 were 1.67E-06 mol/L, 5.36E-06 mol/L, and 2.76E-06 mol/L, respectively, which were significantly lower than that of the Z_WT_ affibody (1.34E-01 mol/L). By contrast, the association rate constants (ka) of the three affibody molecules were significantly higher than that of Z_WT_ affibody **(**Table 1**)**. SPR data clearly show that all three Z_LMP2A-N_ affibodies selected in this study bind to recombinant LMP2A-NCD with high affinity.

**Table 1:** Kinetic data from the SPR biosensor analysis of the affibody molecules.

	Ka(1/Ms)	Kd(1/s)	KD(M)
Z_LMP2A-N_85	2.17E+03	3.63E−03	1.67E−06
Z_LMP2A-N_110	8.21E+02	4.40E−03	5.36E−06
Z_LMP2A-N_252	1.26E+03	3.49E−03	2.76E−06
Z_WT_	4.45E−04	5.96E−05	1.34E−01

*Ka* Association rate constant, *Kd* Dissociation rate constant, *KD* Dissociation equilibrium constant.

To determine the binding site for the Z_LMP2A-N_ affibodies on LMP2A-NCD, epitope mapping was performed by ELISA using overlapping (10-mer) peptides derived from LMP2A-NCD (amino acid, 1–119). The results showed that all three selected affibody molecules could react with the No. 5, 6, 9 and 10 peptides but failed to react with the remaining peptides tested, which was similar to the anti-LMP2A-NCD polyclonal rabbit antibody prepared in our laboratory. As expected, Z_WT_ did not react with any of the peptides tested. Sequence alignment analysis of the No. 5, 6, 9 and 10 peptides revealed that these four peptides shared the common sequence PPPPY, which indicated that the Z_LMP2A-N_ affibodies may bind to the poly-proline (PPPPY) motif in LMP2A-NCD (Fig. 2e).

### Z_LMP2A-N_ affibodies interacted with LMP2A-NCD with high binding specificity

**Fig. 3:**
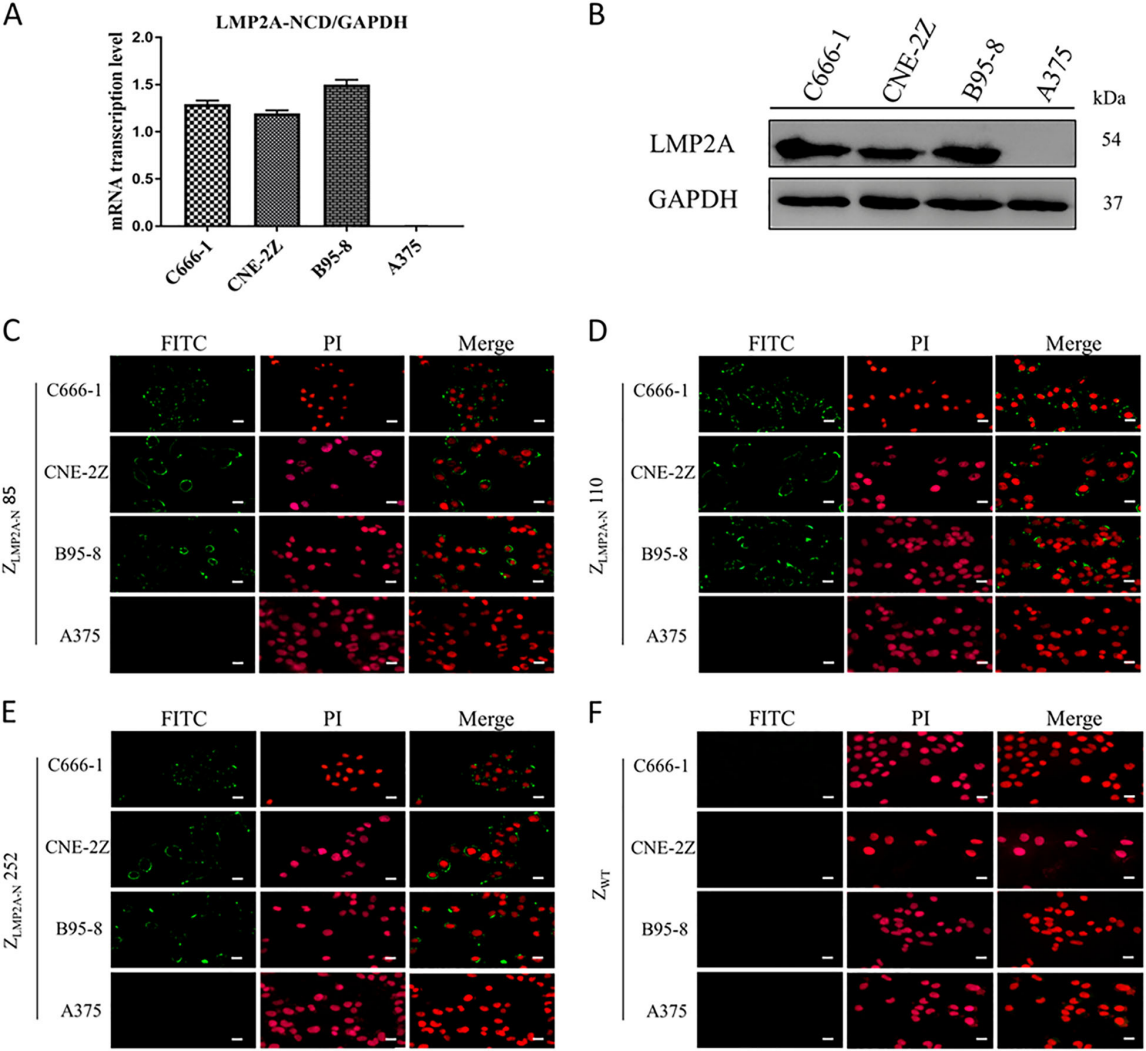
Analysis of the binding specificity of the Z_LMP2A-N_ affibodies for native LMP2A-NCD by an indirect immunofluorescence assay. qRT-PCR (**a**) and Western blotting (**b**) analysis of LMP2A-NCD in C666-1, CNE-2Z, B95-8 cells (EBV-positive) and A375 cancer cells (EBV-negative). Data are given as the mean ± SD (*n* = 3). Images (×400) showing C666-1, CNE-2Z, B95-8 and A375 cells stained with Z_LMP2A-N_85 (**c**), Z_LMP2A-N_110 (**d**) and Z_LMP2A-N_252 (**e**). Z_WT_ affibody was used as a negative control (**f**). Affibody molecules binding to cells are shown in green, while nuclear staining by PI is shown in red. Scale bar = 10 μm.

To assess the interaction of the Z_LMP2A-N_ affibodies with native LMP2A-NCD, we first examined the expression of LMP2A-NCD in EBV-positive cell lines by qRT-PCR and Western blotting. qRT-PCR analysis revealed that LMP2A-NCD mRNA was only expressed in the EBV-positive cell lines (C666-1, CNE-2Z and B95-8) (Fig. 3a), and this result was further confirmed by Western blotting analysis as shown in Fig. 3b. Given that the Z_LMP2A-N_ affibodies were able to bind to recombinant LMP2A-NCD in the SPR analysis, we next investigated whether the Z_LMP2A-N_ affibodies could also specifically bind to native LMP2A-NCD in EBV-positive cells using an indirect immunofluorescence assay (IFA). All three Z_LMP2A-N_ affibodies worked very well as detection reagents and resulted in juxtamembrane region staining in C666-1, CNE-2Z and B95-8 (EBV-positive) cells but not in A375 (EBV-negative) cells (Fig. 3c–e). This staining pattern was similar to the pattern of anti-LMP2A-NCD polyclonal antibody staining (Supplementary Fig. 2), which was used as a positive control. As expected, neither EBV-positive nor EBV-negative cells showed any fluorescence signal when the cells were stained with Z_WT_ affibody (Fig. 3f).

**Fig. 4:**
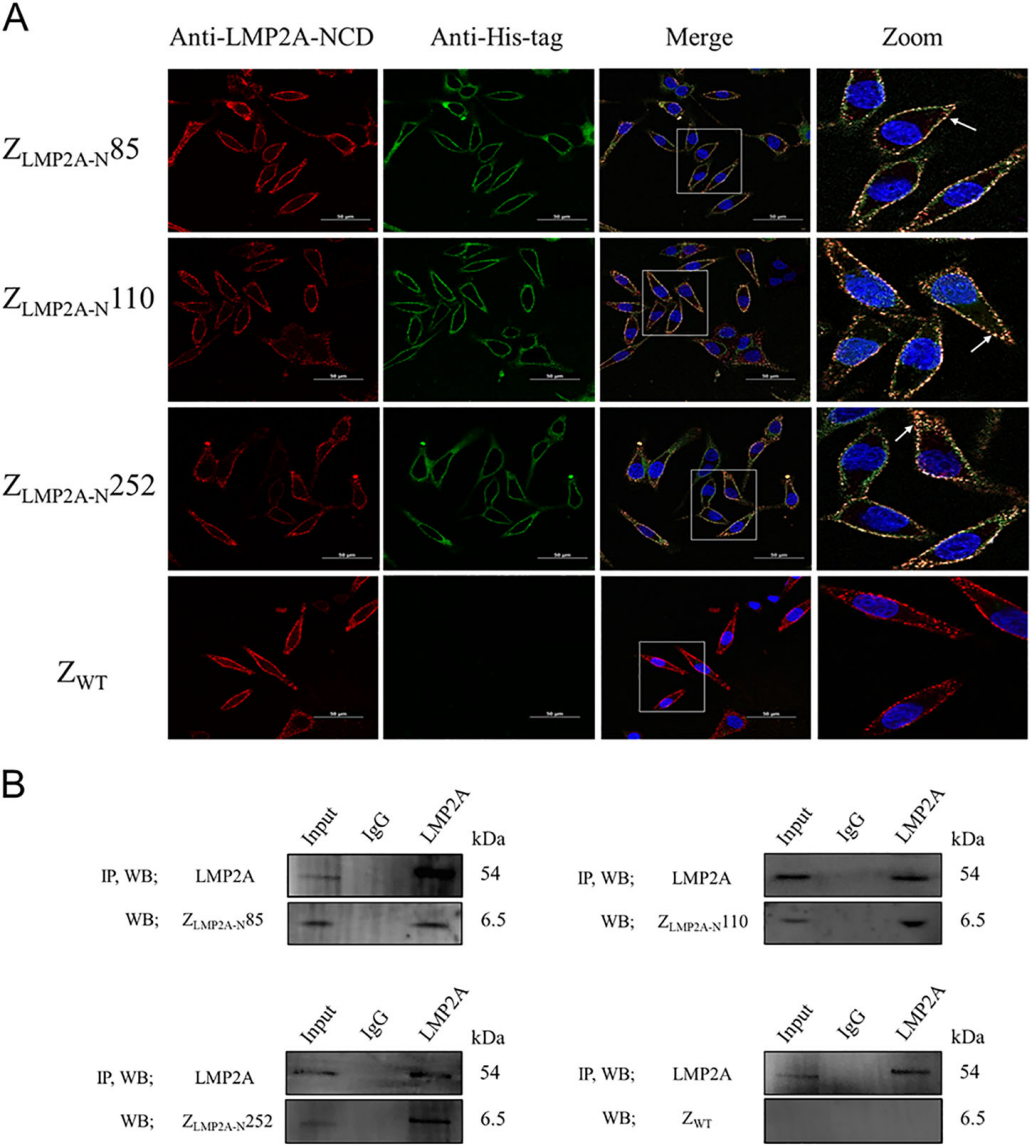
The Z_LMP2A-N_ affibodies interacted with native LMP2A-NCD in the intracellular membranes of C666-1 cells. **a** C666-1 cells were incubated with the Z_LMP2A-N_ affibodies or Z_WT_ control for 3 h and analysed by an indirect immunofluorescence assay. The rat anti-LMP2A mAb (recognise N-terminal domain of LMP2A) and mouse anti-His-tag mAb were used as primary antibodies. The goat anti-rat antibody conjugated with Cy3 (red) and goat anti-mouse antibody conjugated with FITC (green) were used as secondary antibodies. The cell nuclei were stained by Hoechst 3342 (blue). The merge images show the co-localisation of the Z_LMP2A-N_ affibodies with LMP2A-NCD (Yellow). Scale bar = 50 μM. **b** The Z_LMP2A-N_ affibodies form a complex with LMP2A in C666-1 cells that can be detected following LMP2A immunoprecipitation (IP) and subsequent Western blotting analysis with the rat anti-LMP2A mAb or mouse anti-His-tag mAb. In each experiment, lysates incubated with control IgG served as negative controls.

Confocal double immunofluorescence assays and co-immunoprecipitation (co-IP) experiments were performed to further verify the specific binding of the Z_LMP2A-N_ affibodies to the intracellular target. As shown in Fig. 4a, in C666-1 cells, the fluorescence signals of LMP2A-NCD and Z_LMP2A-N_ affibodies were co-localised. Meanwhile, the co-IP assay provided further evidence for a direct interaction between the Z_LMP2A-N_ affibodies and LMP2A. The endogenously expressed LMP2A protein was complexed with the Z_LMP2A-N_ affibodies, followed by IP with an anti-LMP2A antibody (Fig. 4b). Taken together, these data revealed that the Z_LMP2A-N_ affibodies could be internalised into live cells and exhibit strong specific binding to native LMP2A-NCD expressed in EBV-positive cell lines.

### Tumour targeting ability of Z_LMP2A-N_ affibodies in tumour-bearing nude mice

**Fig. 5:**
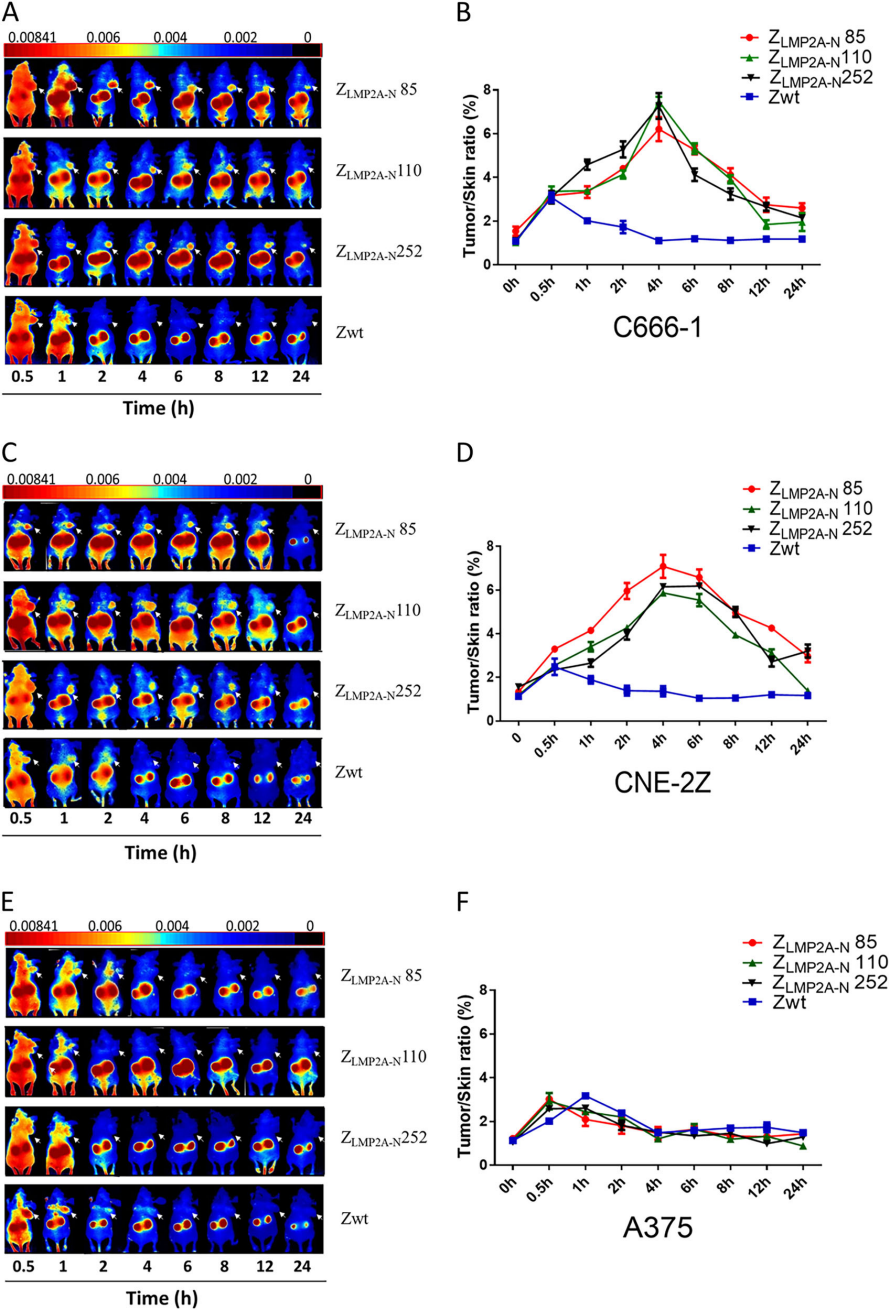
Tumour imaging in model mice by using fluorescence-labelled affibody molecules. Tumour-bearing nude mice (arrows) were generated with cell lines C666-1 (**a**), CNE-2Z (**c**), and A375 (**e**). NIR-based imaging was performed at different time points p.i. with Dylight 755-labelled Z_LMP2A-N_ affibodies. Dylight 755-labelled Z_WT_ affibody was used as a negative control. Tumour/skin ratios were calculated at various time points p.i. of the indicated agents in tumour-bearing mice generated with cell lines C666-1 (**b**), CNE-2Z (**d**), and A375 (**f**). Data are displayed as the mean ± SD (*n* = 3).

We then focused on the biodistribution and imaging characteristics of Dylight 755-labelled affibody molecules in vivo. Athymic nude mice bearing C666-1, CNE-2Z (EBV positive), or A375 (EBV negative) xenografts received an intravenous injection of Dylight 755-labelled Z_LMP2A-N_ affibodies or Z_WT_ affibody. Scanning was performed at different time points p.i. using the NIR imaging system. In the C666-1 and CNE-2Z xenograft models, we observed that high-contrast fluorescence signals of tumour locations occurred at 1 hpi, peaked at 4 hpi, and remained for over 24 h in C666-1 xenografts and for over 12 h in CNE-2Z xenografts (Fig. 5a–d). As expected, no visible fluorescence signal was observed at the tumour location of A375 xenografts **(**Fig. 5e, f). High-contrast imaging effects indicated that all three Z_LMP2A-N_ affibodies have great potential as molecular probes in EBV-related NPC.

In addition, after injection of Dylight 755-labelled Z_WT_ affibody, similar tumour-specific fluorescence signals were not detected in either EBV-positive or EBV-negative xenograft locations (Fig. 5a, c, e). These data provide further evidence that all three Z_LMP2A–N_ affibodies are highly specifically recognised and bind to LMP2A-NCD in EBV xenografts in vivo. As a control group, normal athymic nude mice without tumour xenografts received a tail vein injection of Dylight 755-labelled affibody molecules. We observed non-specific accumulation of the fluorescence signal in the kidney, which indicated that Dylight 755-labelled affibody molecules were cleared by kidney filtration (Supplementary Fig. 3).

### Z_LMP2A-N_ affibodies suppressed EBV-positive NPC cell proliferation by arresting the cell cycle at the G0/G1 phase

**Fig. 6:**
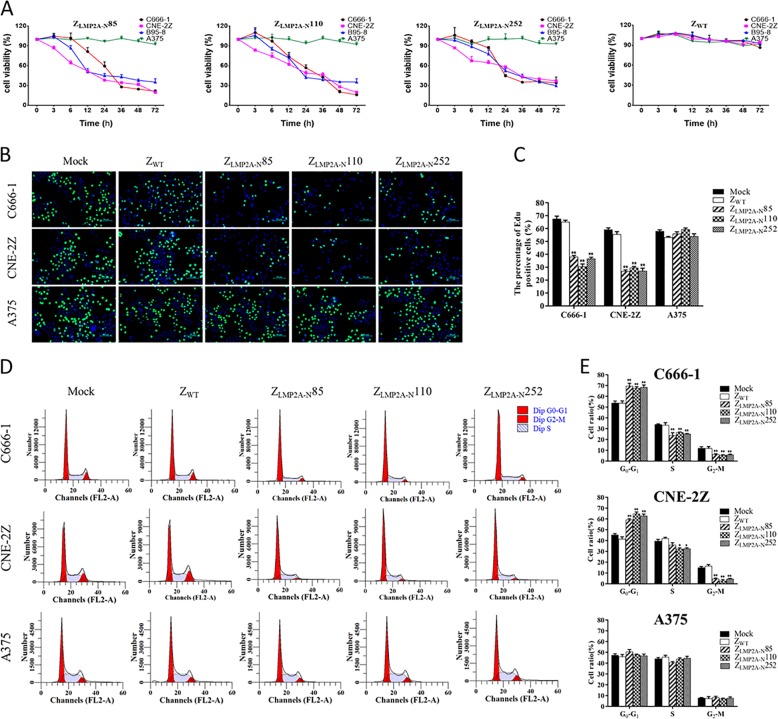
Z_LMP2A-N_ affibodies inhibited cell proliferation and cell cycle progression in EBV-positive cells. **a** CCK-8 assays were performed to determine the viability of EBV-positive cells treated with 10 μM Z_LMP2A-N_ affibodies for the indicated times. EBV-positive (C666-1, CNE-2Z and B95-8) cell viability decreased with increasing incubation time with Z_LMP2A-N_85, Z_LMP2A-N_110 and Z_LMP2A-N_252 compared with the Z_WT_ control, whereas EBV-negative melanoma A375 cells treated with the affibody molecules for the same times remained fully viable. **b** and **c**; EdU staining (**b**) and relative EdU-positive cell ratios (**c**) for cells (C666-1, CNE-2Z and A375) treated with 10 μM Z_LMP2A-N_ affibodies or Z_WT_ control for 24 h. The values are shown as the mean ± SD in three wells. *P < 0.05, **P < 0.01. **d** and **e**; C666-1, CNE-2Z and A375 cells were treated with 10 μM Z_LMP2A-N_ affibodies or Z_WT_ control for 24 h, and the cell cycle was analysed by PI staining and flow cytometry. Representative histograms are shown (**d**), and the percentages of cells in G0/G1, S and G2/M phases were calculated from three independent experiments (**e**). *P < 0.05, **P < 0.01.

CCK-8 assays were performed to evaluate the efficacy of the Z_LMP2A-N_ affibodies in C666-1, CNE-2Z, B95-8 (EBV positive) and A375 cells (EBV negative). After an incubation with Z_LMP2A-N_ affibodies at increasing concentrations for 72 h, the viability of C666-1, CNE-2Z and B95-8 cells was reduced by Z_LMP2A-N_ affibodies in a dose-dependent manner. In contrast, EBV-positive cells treated with Z_WT_ affibody and EBV-negative cells treated with Z_LMP2A-N_ affibodies remained fully viable (Supplementary Fig. 4). After statistical analysis, the half maximal inhibitory concentration (IC50) values for Z_LMP2A-N_85, Z_LMP2A-N_110, and Z_LMP2A-N_252 in C666-1 cells were 9.873 μM, 11.335 μM, and 13.825 μM, respectively. In CNE-2Z cells, these values were 8.735 μM, 11.556 μM and 14.365 μM, respectively. In B95-8 cells, these values were 11.766 μM, 7.876 μM, and 8.322 μM, respectively. According to the IC50 values, the concentration of 10 µM was selected for further investigation. The efficacy of Z_LMP2A-N_ affibodies was evaluated over a time course (0, 3, 6, 12, 24, 36, 48 and 72 h). After incubation with Z_LMP2A-N_ affibodies for the indicated time periods, 10 μM Z_LMP2A-N_ affibodies significantly reduced the viability of C666-1, CNE-2Z and B95-8 cells in a time-dependent manner, whereas Z_WT_ affibody had no effect on any type of cell tested (Fig. 6a). In addition, the results of 5-ethynyl-2-deoxyuridine EdU(green)/Hoechst(blue) immunostaining showed that incubation with 10 μM Z_LMP2A-N_ affibodies for 24 h remarkably weakened the proliferation capacity of EBV-positive cells but not EBV-negative cells, further supporting the ability of the Z_LMP2A-N_ affibodies to effectively inhibit EBV-positive NPC cell proliferation (Fig. 6b, c).

To reveal the underlying mechanisms of the suppressive effect of targeting LMP2A-NCD on EBV-positive NPC cell proliferation, we analysed the percentage of cells in the different phases of the cell cycle by FACS analysis. The representative results of flow cytometry analysis are displayed in Fig. 6d. The results of ModFit software analysis suggested that the Z_LMP2A-N_ affibodies caused a remarkable increase in the proportion of cells in G0/G1 phase in both C666-1 and CNE-2Z cells, accompanied by obvious decreases in the proportions of cells in S phase and G2/M phase (Fig. 6e). Collectively, these results demonstrated that all three Z_LMP2A-N_ affibodies specifically and significantly inhibited cell proliferation and induced cell cycle arrest of their target cells with no detectable cytotoxic effects on other unrelated cells.

### Affibody Z_LMP2A-N_110 supresses β-catenin nuclear translocation in NPC cell lines

**Fig. 7:**
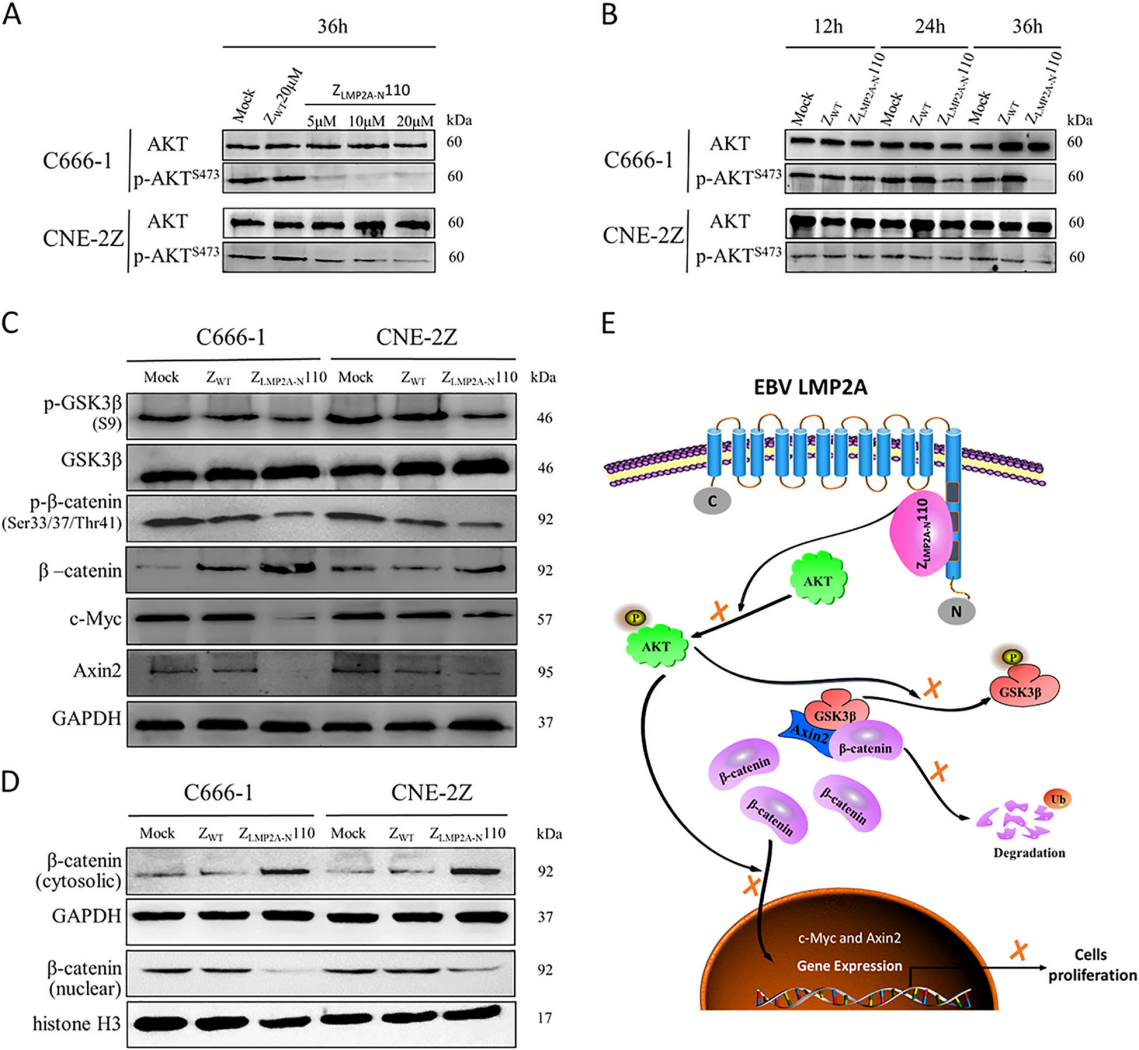
Affibody Z_LMP2A-N_110 inhibited AKT phosphorylation to suppress β-catenin nuclear translocation. p-AKT^(S473)^ was downregulated in a dose-dependent (**a**) and time-dependent (**b**) manner by treatment with affibody Z_LMP2A-N_110 in C666-1 and CNE-2Z cells. **c** p-GSK-3β^(S9)^, p-β-catenin^(Ser33/37/Thr41)^ and downstream effectors of β-catenin signalling, including c-Myc and Axin2, were downregulated, whereas β-catenin levels were upregulated by treatment with Z_LMP2A-N_110 in C666-1 and CNE-2Z cells. **d** Affibody Z_LMP2A-N_110 suppressed the nuclear translocation of β-catenin and promoted the accumulation of β-catenin in the cytoplasm in C666-1 and CNE-2Z cells. The affibody Z_WT_ and medium groups were set as controls. GAPDH or histone served as an internal reference standard. **e** A schematic of the potential model of Z_LMP2A-N_110 blocking the LMP2A-NCD-mediated signalling pathways involving AKT/GSK-3β/β-catenin.

As the poly-proline (PPPPY) motif of LMP2A-NCD is the possible domain recognised by Z_LMP2A-N_ affibodies, we first focused on the AKT/GSK-3β/β-catenin pathway, which is closely related to PPPPY motif and NPC cell proliferation^26–28^, to further investigate the potential molecular mechanisms underlying the cell viability reduction induced by the affibody Z_LMP2A-N_110. Western blotting results revealed that the level of p-AKT^(S473)^ decreased in a concentration-dependent (Fig. 7a) and time-dependent (Fig. 7b) manner in C666-1 cells treated with Z_LMP2A-N_110 compared to the control (mock and Z_WT_). Based on the above results, C666-1 cells treated with 10 µM Z_LMP2A-N_110 for 36 h were selected to detect AKT downstream effectors, including GSK-3β, β-catenin, c-Myc and Axin2. Interestingly, targeting LMP2A-NCD decreased the expression of p-GSK-3β^(S9)^, p-β-catenin^(S33/37/Thr41)^ and downstream factors of β-catenin signalling (c-Myc and Axin2) but increased the expression of β-catenin (Fig. 7c). We further measured the β-catenin level in cytoplasmic and nuclear extracts of C666-1 cells following Z_LMP2A-N_110 treatment by Western blotting and the result revealed that β-catenin was substantially accumulated in the cytoplasm only, whereas the level of nuclear β-catenin was suppressed **(**Fig. 7d**)**. In addition, the data obtained from NPC-derived CNE-2Z cells further confirmed the above results.

## Discussion

Since affibodies were introduced as an alternative to antibodies in the 1990s, a series of affibody molecules have been reported. These affibody molecules are highly suited for vastly different medical applications, including targeted delivery of various payloads, inhibition of peptide aggregation, blocking protein interactions, and molecular imaging diagnosis of tumours^20,21^. More recently, the first therapeutic affibody (ABY-035) against IL-17 was used for clinical trials and was demonstrated to be safe, i.e., non-toxic and non-immunogenic, and well tolerated in 25 patients and 46 healthy volunteers^21^ (www.affibody.se). Due to their small size (~6.5 kDa), which is less than one-twentieth of an antibody and one-fourth of a single-chain fragment variable (scfv), affibody molecules can be easily produced by recombinant gene expression or conventional peptide synthesis methods. In this study, we executed panning, ELISA screening and DNA sequencing from a phage display library to obtain three potential LMP2A-NCD-binding affibody molecules (Z_LMP2A-N_85, Z_LMP2A-N_110 and Z_LMP2A-N_252). We then produced these affibodies in a prokaryotic expression system and further verified their affinity and specificity for the target protein. Kinetic BIAcore analysis indicated that each of the Z_LMP2A-N_ affibodies bound to LMP2A-NCD with affinities approximately 10^5^ times higher than that of Z_WT_ and reached the μM level, in accordance with previous studies of Z_HPV16E7_384^25^, Z_HPV16E7_384-Z_HPV18E7_228^29^, and Z_HPV16E7_ affitoxin384^30^ in our laboratory. Furthermore, according to IFA, the Z_LMP2A-N_ affibodies could specifically bind to EBV-positive cell lines, and LMP2A-NCD and Z_LMP2A-N_ affibodies were also co-localised in the juxtamembrane region. In addition, the co-IP assay provided further evidence for a direct subcellular interaction between the Z_LMP2A-N_ affibodies and LMP2A. More importantly, in tumour-bearing nude mice, Z_LMP2A-N_ affibodies are capable of target-specific accumulation in EBV xenografts. All of these results indicate that the Z_LMP2A-N_ affibodies can bind to LMP2A-NCD with high affinity and specificity both in vitro and in vivo.

Molecular imaging in cancer diagnosis can provide critical information for a global view of potential metastatic lesions and contribute qualitative information regarding preclinical disease that is currently unavailable by conventional imaging techniques^21^. However, diagnosis based on molecular imaging has not been widely adopted in NPC, partly due to the lack of suitable targeting probes with high target-binding affinity and specificity. Therefore, developing an ideal probe for NPC diagnosis has attracted much attention. mAbs are a straightforward way to develop imaging agents. However, mAbs have the intrinsic limitation of a long residence time in circulation, leading to poor imaging contrast, and the analysis must be performed several days p.i., which limits its application to some extent^20,21^. Recent reviews have described in detail that ideal probes should be as small as possible and have high affinity, rapid biodistribution and tissue penetration, enrichment of the local concentration within a short period of time, and rapid clearance of unbound tracking agents to provide high-contrast tumour imaging^31^. Affibody molecules represent a novel category of affinity molecules that can provide efficient tumour penetration, high-affinity cancer-specific ligands and cost-efficient production. In addition, affibody molecules have been developed and successfully applied as imaging tracers to detect HER2-overexpression tumours in both preclinical and clinical studies^32–34^. In a previous study, one HER2-binding affibody molecule, Z_HER2_342, had better tumour uptake and provided higher tumour-to-blood ratios than did HER2-binding scFv antibody fragments in the imaging of HER2-expressing SKOV-3 xenografts^22^. Similar to observations in previous studies^22,33^, we also detected Dylight 755-labelled Z_LMP2A-N_ affibodies in the tumour position of EBV-positive mice as early as 1 hpi in this study. These affibodies rapidly accumulated for clear, high-contrast tumour imaging within 4 h and were retained in tumours for over 24 h. The biodistribution data showed that Z_LMP2A-N_ affibodies were cleared via renal filtration and were almost completely cleared from the mouse at 48 h. Collectively, these properties strongly implicate the favourability of Z_LMP2A-N_ affibodies for molecular imaging and may improve the accuracy of early diagnosis and, thereby, the prognosis of NPC.

New strategies including molecular-targeted therapy are promising treatment options for NPC, especially for patients who have local recurrence and distant metastases^18,35^. Although mAbs such as cetuximab and bevacizumab have yielded impressive cancer control effects in preclinical studies of NPC^36,37^, the stability, solubility and size limitations of mAbs have urged researchers to seek complementing strategies. For therapeutic applications solely based on blocking the activity of tumour-related target protein as a mechanism of action, the fragment crystallisable (Fc) region of mAbs mediating, for instance, complement-dependent cytotoxicity and antibody-dependent cell-mediated cytotoxicity (ADCC) is not required and might even be undesired^21^. Similar to non-Fc-portion antibody derivatives, such as scFv, fragment variable (Fv), and fragment antigen binding (Fab), affibody molecules are very valuable alternatives for such indications, and several affibody-based tumour targeting therapeutic agents with robust biological activity and no immunogenicity issues have been reported^30,38,39^. Affibody molecules do not contain any disulphide bridges, an observation suggesting that they could fold in the reducing environment of the cell cytoplasm^40^. This could allow for intracellular applications such as in vivo antitumor therapy using an affibody targeting HPV16E7^30^. In our work, given that the 119 amino acids of the LMP2A N-terminal cytoplasmic domain could constitutively activates many kinases and signal transduction molecules involved in cell cycle, proliferation, survival and motility^3^, we investigated the effect of the selected LMP2A-NCD-binding affibodies on EBV-positive NPC cells and explored the underlying mechanism. We exposed three EBV-positive cell lines to Z_LMP2A-N_ affibodies; in all three cell lines, targeting LMP2A-NCD by Z_LMP2A-N_ affibody treatment significantly reduced cell viability in a time-dependent and concentration-dependent manner. Subsequent studies verified that the reduction in cell proliferation induced by treatment with the Z_LMP2A-N_ affibodies could probably be attributed to cell cycle arrest in the G0/G1 phase in EBV-positive cells. Because the epitope mapping experiments suggested that the Z_LMP2A-N_ affibodies can bind to the PPPPY motif, which is critical for the activation of β-catenin signalling^3,26^, we further investigated the expression and phosphorylation of AKT/GSK-3β/β-catenin signalling proteins. The Western blotting results showed that treatment with the affibody Z_LMP2A-N_110 significantly decreased the expression of p-AKT^(Ser473)^, p-GSK-3β^(Ser9)^, p-β-catenin^(Ser33/37/Thr41)^ and factors downstream of β-catenin, including c-Myc and Axin2, but increased the expression of β-catenin. Given that the activity of β-catenin signalling relies on the translocation and accumulation of β-catenin in the nucleus^41–43^, we used Western blotting to further measure the β-catenin levels in cytoplasmic and nuclear extracts of NPC-derived cells treated with Z_LMP2A-N_110. The result showed that β-catenin was substantially accumulated in the cytoplasm only. In contrast, β-catenin significantly decreased in the nucleus, leading to inhibition of the downstream effector c-Myc, an important participant in cell proliferation^44^. Therefore, our results suggest that c-Myc expression downregulation, which followed β-catenin nuclear translocation suppression, is a potential factor responsible for the NPC-derived cell viability reduction.

In summary, we generated three novel Z_LMP2A-N_ affibodies (Z_LMP2A-N_85, Z_LMP2A-N_110 and Z_LMA-N_252) and confirmed their high affinity and specificity for the LMP2A N-terminal cytoplasmic domain through SPR, indirect immunofluorescence, co-IP assays and NIR small animal fluorescence imaging detection in vitro and in vivo. The detailed mechanism underlying the reduction of NPC-derived cell viability by Z_LMP2A-N_ affibodies in vivo remains to be further investigated. Nevertheless, we showed that affibody Z_LMP2A-N_110 could inhibit phosphorylation of AKT, GSK-3β and β-catenin, leading to suppression of β-catenin nuclear translocation and ultimately inhibition of the expression of the c-Myc oncogene. Therefore, these novel Z_LMP2A-N_ affibodies will be a potent molecular imaging probe and targeted therapeutic agent that might be useful for improving the early diagnosis and clinical outcome of NPC patients.

## Methods

### Materials

Escherichia *coli* BL21(DE3), pET21a(+) vector, and phagemid vector pCANTAB5E were purchased from American Type Culture Collection (ATCC), Novagen, and Amersham Pharmacia Biotech, respectively. The reagents used included helper phage M13K07 (New England Biolabs, MA, USA), restriction endonucleases (S*fi I*, N*ot I*, N*de*I and X*ho*l) and T4 DNA Ligase (Thermo Fisher Scientific, MA, USA), glutathione-agarose column, thrombin and horseradish peroxidase (HRP)/anti-M13 monoclonal antibodies (GE Healthcare, Uppsala, Sweden), isopropyl-D-thiogalactopyranoside (IPTG), paraformaldehyde and Triton X-100 (Sigma Aldrich, Saint Louis, USA), Ni-NTA agarose (Qiagen, Valencia, CA), Protein A+G Agarose and a bicinchoninic acid (BCA) kit (Beyotime, Beijing, China). Roswell Park Memorial Institute 1640 (RPMI-1640), Dulbecco’s Modified Eagle’s Medium (DMEM), trypsin-ethylenediaminetetraacetic acid (EDTA), foetal bovine serum (FBS), penicillin, and streptomycin were obtained from Gibco. TRIzol reagent, reverse transcription kits, and qPCR master mix were obtained from Takara Biomedical Technology Co., Ltd. (Beijing, China). Goat anti-mouse antibody conjugated to fluorescein isothiocyanate (FITC), goat anti-rabbit antibody conjugated to FITC, goat anti-rat antibody conjugated to Cy3, propidium iodide (PI), 3,5,3′,5′-tetramethylbenzidine (TMB) solutions and the EdU labelling/detection kit were purchased from MultiSciences Biotech Co., Ltd. (China). The reagents used in the study also included Dylight 755 (Thermo Fisher Scientific, MA, USA), Cell Counting Kit-8 (CCK-8) (Dojindo, Kumamoto, Japan), cell lysis buffer (Beyotime, Beijing, China), and a nuclear-cytosol extraction kit (Applygen, Beijing, China). Protease inhibitors and phosphatase inhibitors were purchased from Roche. Rabbit immune serum anti-EBV LMP2A N-terminal recombinant proteins were prepared in our laboratory. All primary and secondary antibodies used in Western blotting assays were obtained from Abcam or Cell Signalling Technology.

### Construction of a Z domain combinatorial library

A combinatorial phage library of the Z domain was prepared as described previously^25^ with random amino acid residues at positions 9, 10, 11, 13, 14, 17, 18, 24, 25, 27, 28, 32 and 35. Briefly, according to the amino acid sequence and structure of the Wild SPA-Z scaffold (Z_WT_), random primers were designed to target the coding sequences corresponding to the three helix structural domains. An SPA sequence that could cause random amino acid changes was amplified by polymerase chain reaction (PCR) and named SPA-N. Using routine molecular cloning methods, the SPA-N coding sequence was cloned into a phagemid (pCANTAB5E) using the S*fi I* and N*ot I* sites to construct the pCANTAB5E/SPA-N recombinant phagemid, which was transformed into competent *E. coli* TG1(DE3). The capacity of the natural affibody library cloned into the vector was approximately 1 × 10^8^, and this library had 100% diversity in the SPA-Z scaffold. After evaluating the capacity and randomness of the inserted affibody library, the phage stocks were used to pan potential affibodies that specifically bind to LMP2A-NCD with high affinity by using phage display technology.

### Production of recombinant LMP2A-NCD protein

Preparation of highly purified LMP2A-NCD protein was performed according to the protocols established in our lab^45^. Briefly, the sequence of LMP2A-NCD containing six histidines at its C-terminus was subcloned into the glutathione S-transferase (GST) gene fusion vector pGEX-4T-1. The fusion protein, GST-LMP2A-NCD-His6-tagged, was expressed in *E. coli* BL21 (DE3) and purified by affinity chromatography on a glutathione-agarose column. After digestion with thrombin, the LMP2A-NCD-His6-tagged fragment was further purified on a Ni-NTA Sepharose column and verified by sodium dodecyl sulfate polyacrylamide gel electrophoresis (SDS-PAGE) and a Western blotting assay using an anti-His-tag mAb (Supplementary Fig. 1A).

### Selection of potential affibodies binding to LMP2A-NCD with high affinity

Highly purified LMP2A-NCD protein was used as a targeted protein during three rounds of phage display panning and enzyme-linked immunosorbent assay (ELISA) selections for target-binding activity. Screening for potential affibody molecules binding to LMP2A-NCD was performed as described in a previous study^25^. After panning, ELISA screening and DNA sequencing, the sequences of inserted fragments in selected phages acted as potential affibodies with high affinity and specifically bound to the recombinant protein.

### Expression and purification of Z_LMP2A-N_ affibodies

Gene fragments encoding the selected affibody molecules Z_LMP2A-N_85, Z_LMP2A-N_110, and Z_LMP2A-N_252, as well as Z_WT_, were sub-cloned into the N*de* I and X*ho* I restriction sites of the pET21a(+) expression vector. Following confirmation by DNA sequencing, positive plasmids were transformed into *E. coli* BL21 (DE3) for expression of the fusion proteins. The His6-tagged recombinant protein was induced by 1 mM IPTG for 6 h at 37 °C and purified by a Ni-NTA Sepharose column according to the manufacturer’s recommendations. Purified proteins were verified by SDS-PAGE and further confirmed by a Western blotting assay using an anti-His-tag mAb. After determining the concentration using the BCA protein quantitation method, the purified proteins were stored at −80 °C for future use.

### Biosensor interaction analysis

Surface plasmon resonance (SPR) was performed on a BIAcore T200 (GE Healthcare, Uppsala, Sweden) to assess the interaction between the Z_LMP2A-N_ affibodies and LMP2A-NCD. The highly purified protein served as a target ligand and was immobilised on the surface of Sensor Chip CM5 (GE Healthcare). Various concentrations of each analyte sample (Z_LMP2A-N_ affibodies) were prepared ranging from 2.5–20 μM to flow over the chip and to characterise its interaction with the immobilised ligand. Z_WT_ affibody was set as a negative control. The resulting sensorgrams were fit globally using a one-to-one Langmuir binding model and analysed by BIAcore T200 evaluation 3.0.2 software.

### Epitope mapping by peptide-coated ELISA

Synthetic peptides (Shanghai Bootech BioScience & Technology Co., Ltd) were dissolved in coating buffer and immobilised on 96-well plates at 10 μg/ml. After blocking with Blocking Buffer (phosphate-buffered saline with Tween 20 containing 5% skim), the plates were incubated with purified Z_LMP2A-N_ affibodies (100 μg/ml), followed by anti-His-tag-mouse IgG. After washing with phosphate-buffered saline with Tween 20 (PBST), the bound antibodies were detected after incubation with HRP-conjugated anti-mouse IgG. Subsequently, the plates were washed with PBST and incubated with TMB substrate for 15 min at ambient temperature before adding 1 M H_2_SO_4_ to terminate the reaction. A microplate reader (BioTek, Winooski, VT, USA) was used to measure the absorbance at 450 nm. All the samples were run in triplicate, and anti-LMP2A-NCD polyclonal antibody was used as a positive control.

### Cell culture

EBV LMP2A-positive cell lines, including C666-1, CNE-2Z (Human NPC cell lines, obtained from Taisheng Bio-Tech Co., Ltd., Guangzhou, China), B95-8 (EBV transformed lymphocyte, ATCC: CRL-1612) and EBV-negative melanoma A375 (ATCC: CRL-1619), were used for evaluation of Z_LMP2A-N_ affibody cytotoxicity and cell binding affinity. C666-1, CNE-2Z and B95-8 cells were cultured in RPMI-1640 medium supplemented with 10% FBS, 100 U/ml penicillin and 100 mg/ml streptomycin, while A375 cells were cultured in DMEM medium supplemented in the same manner as EBV-positive cells. All cells were maintained at 37 °C in a humidified atmosphere containing 5% CO_2_.

### LMP2A-NCD expression in EBV-positive cell lines

C666-1, CNE-2Z, B95-8 and A375 cells were cultured separately in 6-well dishes for 24 h at 37°C. Total RNA was extracted from cells using TRIzol reagent and dissolved in diethylpyrocarbonate (DEPC)-treated water. Total RNA was adjusted to a final concentration of 0.1 μg/ml and reverse-transcribed into cDNA using a reverse transcription kit. cDNA samples were mixed with primer pairs (1. LMP2A-NCD: 5′-CGACCGTCACTCGGACTATCA, 3′-TTCCTCTGCCCGCTTCTTC; 2. GAPDH: 5′-TGAACGGGAAGCTCACTGG, 3′-TCCACCACCCTGTTGCTGTA), and a qPCR Master Mix was prepared for subsequent qPCR analyses using an Applied Biosystems Real Time qPCR System (Thermo Fisher Scientific, MA, USA). The results were analysed by using Real Time qPCR software (QuanStudio 6, Life Technologies).

Western blotting was performed to determine the LMP2A expression levels in tumour cells. After incubation for 24 h, the cells were lysed in lysis buffer. The proteins were separated by 10% SDS-PAGE and blotted onto polyvinylidene difluoride membranes (PVDF; Millipore, Billerica, MA). The blots were blocked with PBST containing 5% skim milk for 2 h at 37 °C, incubated with primary antibody (rabbit anti-LMP2A-NCD antibody, prepared in-house) overnight at 4 °C, and probed with a fluorescent secondary antibody for 1 h. The fluorescence was visualised with a Western Blotting Imaging System (Clinx, Shanghai, China) and analysed with ImageJ 1.33 software (National Institutes of Health). Glyceraldehyde 3-phosphate dehydrogenase (GAPDH) served as an internal reference standard.

### Cell targeting in vitro

An indirect immunofluorescence assay was performed to assess the targeting specificity of Z_LMP2A-N_ affibodies in vitro. C666-1, CNE-2Z, B95-8 and A375 cells were plated evenly on cover slides in 6-well cell culture plates and incubated for 3 h with Z_LMP2A-N_ affibodies or Z_WT_ affibody control at a final concentration of 100 μg/ml. After washing with PBS thrice to remove the free molecules, the cells were then fixed with 4% paraformaldehyde and permeabilised with 0.3% Triton X-100 for 10 min at 37 °C. The cells were then incubated in blocking buffer (RPMI-1640 containing 20% FBS) for 1 h at 37 °C before incubation with primary antibody (mouse anti-His-tag mAb) in blocking buffer overnight at 4 °C, followed by the addition of secondary antibodies FITC-conjugated goat anti-mouse IgG (H + L) at 37 °C for 1 h. Cell nuclei were stained with PI at 37 °C for 5 min. The images were visualised using a confocal fluorescence microscope (Nikon C1-i, Japan).

To further confirm the specific intracellular binding of Z_LMP2A-N_ affibodies to native LMP2A-NCD, co-localisation in C666-1 cells was assessed by a confocal double immunofluorescence assay. The procedure was similar to the above description.

### Immunoprecipitation

C666-1 cells were plated evenly in 6-well cell culture plates and incubated for 3 h with the Z_LMP2A-N_ affibodies or Z_WT_ control at a final concentration of 100 μg/ml. After washing, the cells were lysed for 15 min on ice with cell lysis buffer supplemented with protease inhibitors. Then, 5 μg of the rat antibody specific to LMP2A (Abcam, Clone 15F9) was combined with disuccinimidyl suberate bound to protein A/G plus agarose. After washing thoroughly with PBS according to the manufacturer’s instructions, the protein complexes were resuspended in reducing SDS sample buffer, heated at 95 °C for 10 min, and analysed by Western blotting.

### Animal models

All animal experiment protocols were approved by the Ethical Committee of Wenzhou Medical University. C666-1, CNE-2Z and A375 cells (2 × 10^6^) were injected subcutaneously into the upper axillary fossa of nude mice (BALB/c, 4–5 weeks old, *n* = 3 per group) purchased from the Shanghai SLAC laboratory animal CO., LTD (Shanghai, China). When the tumour volume reached 300~500 mm^3^, nude mice were used for near-infrared (NIR) optical imaging.

### Tumour targeting in vivo

The dynamic distribution and tumour targeting ability of Z_LMP2A-N_ affibodies were investigated in nude mice using NIR optical imaging. Affibody molecules were labelled with Dylight 755 according to previously described methods^25^ and then injected (100 μg; 150 μL per mouse) into tumour-bearing nude mice through the tail vein. To verify whether the uptake was mediated by specifically targeting LMP2A-NCD, we used an EBV-negative (A375) xenograft treated with Z_LMP2A-N_ affibodies and an EBV-positive (C666-1 and CNE-2Z) xenograft treated with Z_WT_ affibody as the controls. Each group included at least three mice. An in vivo NIR imaging system (Cri Maestro 2.10, USA) was used for imaging at different time points post-injection (p.i.). In addition, the tumour/skin tissue fluorescence signal intensity ratios at different time points p.i. were analysed.

### Efficacy of Z_LMP2A-N_ affibodies in vitro

Cell viability assays were performed to evaluate the efficacy of the Z_LMP2A-N_ affibodies in C666-1, CNE-2Z, and B95-8 cells (EBV positive) using the CCK-8 kit. Cells were seeded onto 96-well plates at a density of 5 × 10^3^ cells/well (*n* = 3). After 24 h, the cells were treated with Z_LMP2A-N_ affibodies at increasing concentrations (1, 2.5, 5, 10, 20 and 40 μM). EBV-positive cells treated with Z_WT_ affibody and EBV-negative cells (A375) treated with Z_LMP2A-N_ affibodies were set as negative controls. Cell viability was determined after incubation for 0, 3, 6, 12, 24, 36, 48 and 72 h. CCK-8 solution (10 μL) was added to each well and incubated for an additional 30 min. The absorbance of the solution at 450 nm was measured using a microplate reader to analyse cell viability.

### EdU cell proliferation assay

Cell proliferation was analysed by using an EdU labelling/detection kit based on the manufacturer’s protocol. Briefly, the cells were seeded in 24-well plates at 2.5 × 10^4^ cells per well and stored at 37 °C under 5% CO_2_. After incubation with the Z_LMP2A-N_ affibodies for 24 h, 50 μM EdU labelling medium was added to the cells and incubated for an additional 2 h. The cells were treated with 4% paraformaldehyde and then 0.5% Triton X-100 for 10 min each at room temperature. Then, the cells were stained with an anti-EdU working solution and subsequently incubated with 100 μL of Hoechst 33342 (5 μg/ml). The percentage of EdU-positive cells was clearly observed under a fluorescence microscope. The percentage of EdU-positive cells was calculated from five random fields in three wells.

### Cell cycle analysis

C666-1, CNE-2Z and A375 cells were harvested after incubation with the Z_LMP2A-N_ affibodies for 24 h and then fixed with 1 ml of 70% cold ethanol in PBS at 4 °C overnight. The fixed cells were washed with PBS, stained with PI (50 μg/ml) and incubated in the dark for 30 min. The DNA content of the cells was analysed by a FACS Calibur flow cytometer (BD Biosciences, San Jose, CA), and the proportions of cells in the different phases of the cell cycle were assessed using ModFit LT 3.0 software.

### Western blotting analysis for signalling proteins

C666-1 and CNE-2Z (NPC-derived cells) were seeded in 6-well plates (1 × 10^5^) and incubated with medium containing either Z_LMP2A-N_110 or Z_WT_ or medium alone. After various treatments for the indicated periods of time, cells were lysed in cell lysis buffer supplemented with protease inhibitors and phosphatase inhibitors and then quantified by a BCA protein assay kit. Cytoplasmic and nuclear extracts were prepared with a nuclear-cytosol extraction kit in accordance with the manufacturer’s protocol. Equal amounts of protein (30 µg) were separated by 8–12% SDS-PAGE and blotted onto a PVDF membrane. The PVDF membrane was blocked with 5% skim milk in PBST buffer. Membranes were incubated with primary antibodies (Supplementary Table 1) overnight at 4 °C with shaking, followed by incubation with corresponding secondary antibodies at 37 °C for 1.5 h. Fluorescence was visualised with Western Blotting Imaging System and analysed with ImageJ 1.33 software. GAPDH or histone served as an internal reference standard.

### Statistical analysis

Data are presented as the mean ± standard deviation (SD). Statistical analysis of the significance between groups was conducted using Student’s test, and *P* < 0.05 was considered statistically significant. All the calculations were performed with the SPSS16.0 software.

## Supplementary information

Supplementary figure and table legends

Figure S1

Figure S2

Figure S3

Figure S4

Figure S-T1
