# Correction: Generation of novel affibody molecules targeting the EBV LMP2A N-terminal domain with inhibiting effects on the proliferation of nasopharyngeal carcinoma cells

**DOI:** 10.1038/s41419-020-2692-9

**Published:** 2020-06-30

**Authors:** Jinshun Zhu, Saidu Kamara, Danwei Cen, Wanlin Tang, Meiping Gu, Xingyuan Ci, Jun Chen, Lude Wang, Shanli Zhu, Pengfei Jiang, Shao Chen, Xiangyang Xue, Lifang Zhang

**Affiliations:** Institute of Molecular Virology and Immunology, Department of Microbiology and Immunology, School of Basic Medical Sciences, Wenzhou Medical University, 325035 Zhejiang, Wenzhou China

**Keywords:** Tumour virus infections, Tumour virus infections, Diagnostic markers, Diagnostic markers

Correction to: *Cell Death and Disease*

10.1038/s41419-020-2410-7 published online 01 April 2020

The original version of this Article incorrectly gave the foundation number of the Public Welfare Foundation of Zhejiang Province as GF18H160070. The correct number is LGF18H160030. This has now been corrected in the PDF and HTML versions of the Article.

