# Supplementary material for: Generation of novel affibody molecules targeting the EBV LMP2A N-terminal domain with inhibiting effects on the proliferation of nasopharyngeal carcinoma cells
Source: Cell Death Dis. 2020 Apr 1;11(4):213. doi: 10.1038/s41419-020-2410-7 (PMC7113277; doi:10.1038/s41419-020-2410-7)
Supplement: Supplementary file 1 — Supplementary figure and table legends [file 41419_2020_2410_MOESM1_ESM.docx]

**Supplementary Figure S1:** **(A)** SDS-PAGE and Western blotting analysis of the purified recombinant LMP2A-NCD protein. Lane 1, recombinant LMP2A-NCD protein; lane 2, Western blotting analysis of LMP2A-NCD; **(B)** ELISA screening for target-binding activity of potential EBV LMP2A N-terminal domain-binding affibody molecules from 65 clones.

**Supplementary Figure S2: Direct Immunofluorescence staining using anti-LMP2A-NCD antibody**. Immunofluorescence staining of EBV-positive cell lines (C666-1, CNE-2Z and B95-8) and an EBV-negative cell line (A375) with anti-LMP2A-NCD rabbit immune sera, followed by FITC-conjugated goat anti-rabbit secondary antibody. Nuclei were counterstained with PI (red).

**Supplementary Figure S3:** **In vivo biodistribution of affibody molecules in healthy normal mice**. After tail vein injection with Dylight 755-labelled affibody molecules, fluorescence images were obtained from mice at different time points. Kidney uptake was prominent for the accumulation of affibody molecules. The accumulation of affibody molecules maximally occurred at 6 h p.i. and then decreased over the time course. The signal was undetectable at 96 hpi.

**Supplementary Figure S4: Efficacy of Z_LMP2A-N_ affibodies in vitro.** C666-1, CNE-2Z, B95-8 and A375 cells treated with the indicated concentrations of Z_LMP2A-N_85, Z_LMP2A-N_110, Z_LMA-N_252 or Z_WT_ for 72 h. Cell viability was measured using a CCK-8 assay. EBV-positive cell viability decreased with increasing concentrations of Z_LMP2A-N_85, Z_LMP2A-N_110 and Z_LMA-N_252 compared with that with the Z_WT_ control, whereas A375 cells treated with the same concentrations of affibody molecules remained fully viable. Data are displayed as the mean ± SD (n=3).
