# Supplementary figures and images for: Generation of novel affibody molecules targeting the EBV LMP2A N-terminal domain with inhibiting effects on the proliferation of nasopharyngeal carcinoma cells
Source: Cell Death Dis. 2020 Apr 1;11(4):213. doi: 10.1038/s41419-020-2410-7 (PMC7113277; doi:10.1038/s41419-020-2410-7)

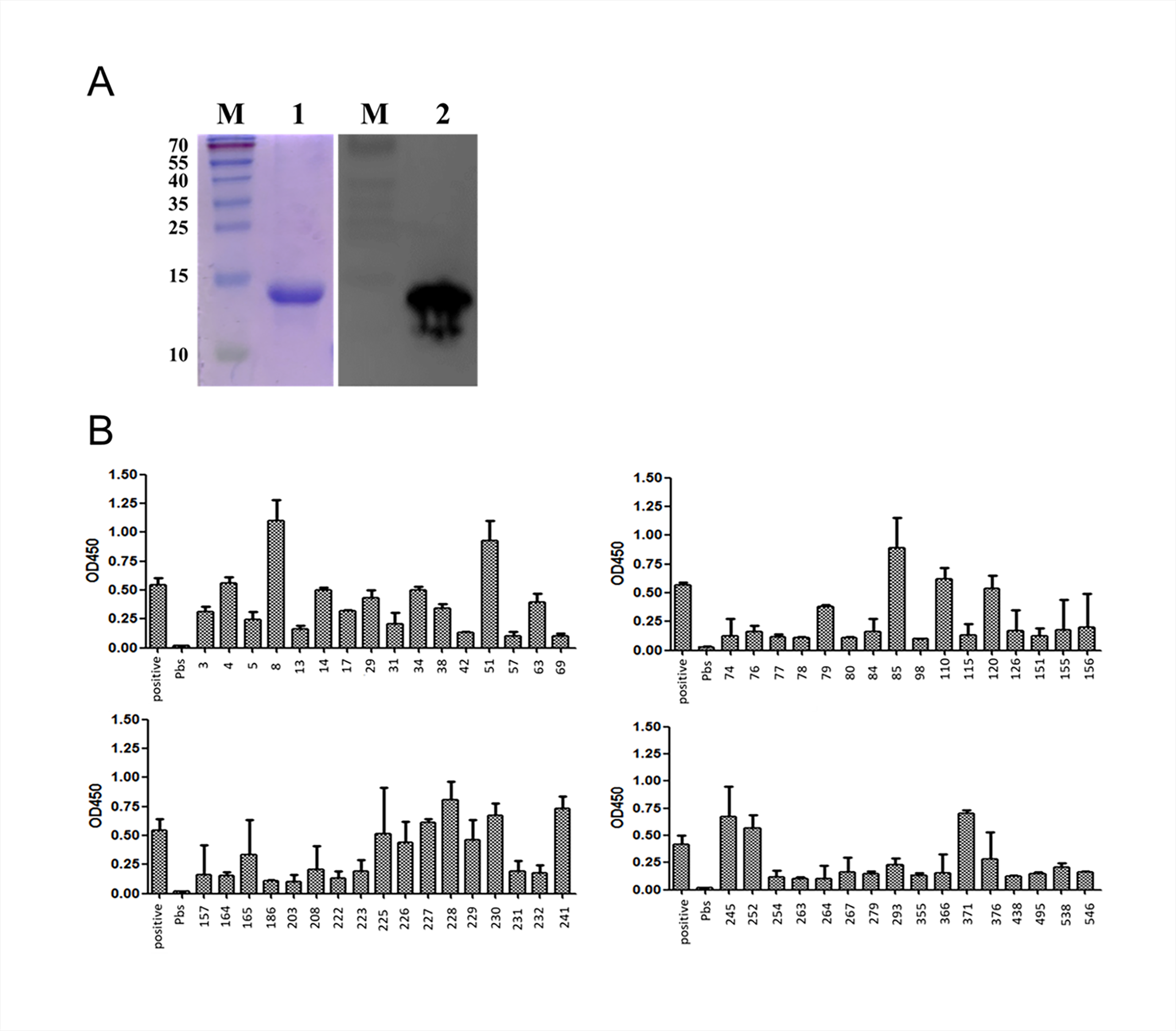

Supplement: Supplementary file 2 — Figure S1 [file 41419_2020_2410_MOESM2_ESM.tif]

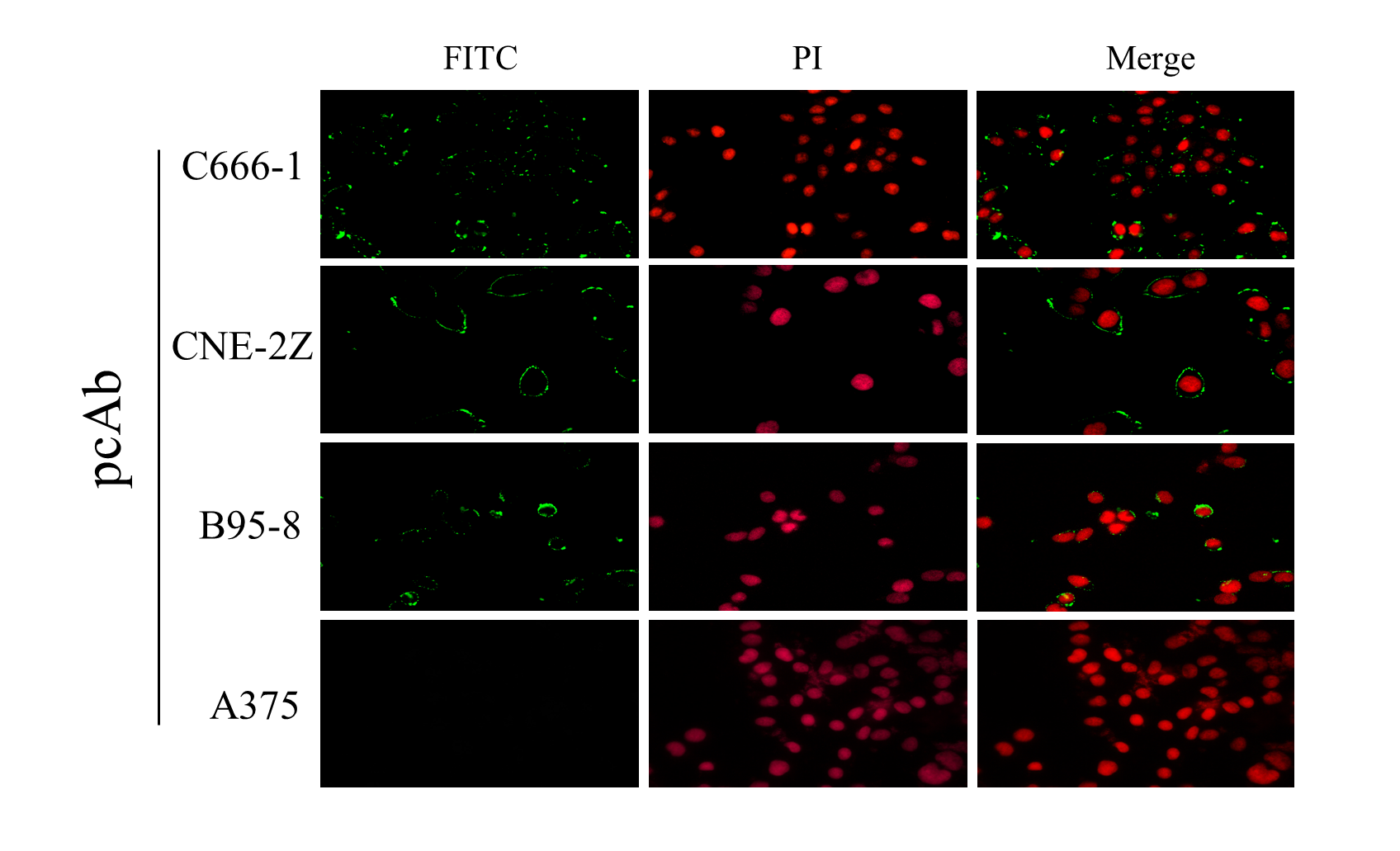

Supplement: Supplementary file 3 — Figure S2 [file 41419_2020_2410_MOESM3_ESM.tif]

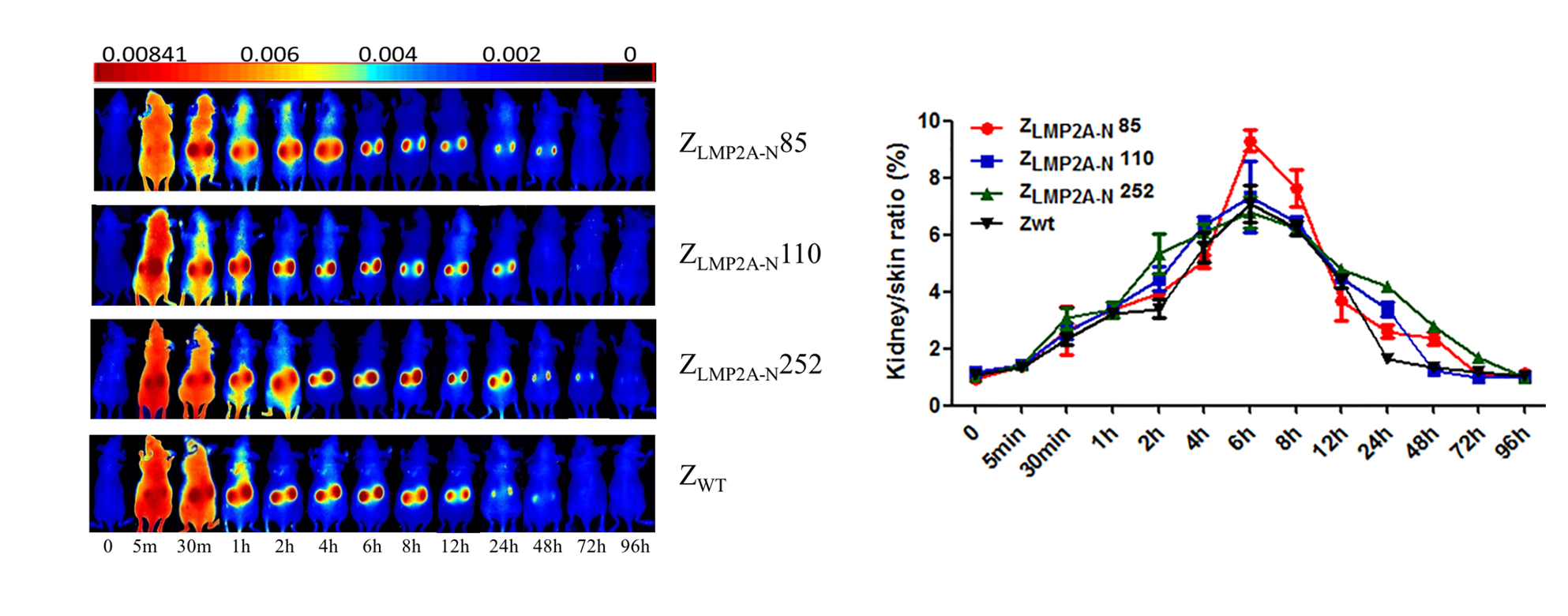

Supplement: Supplementary file 4 — Figure S3 [file 41419_2020_2410_MOESM4_ESM.tif]

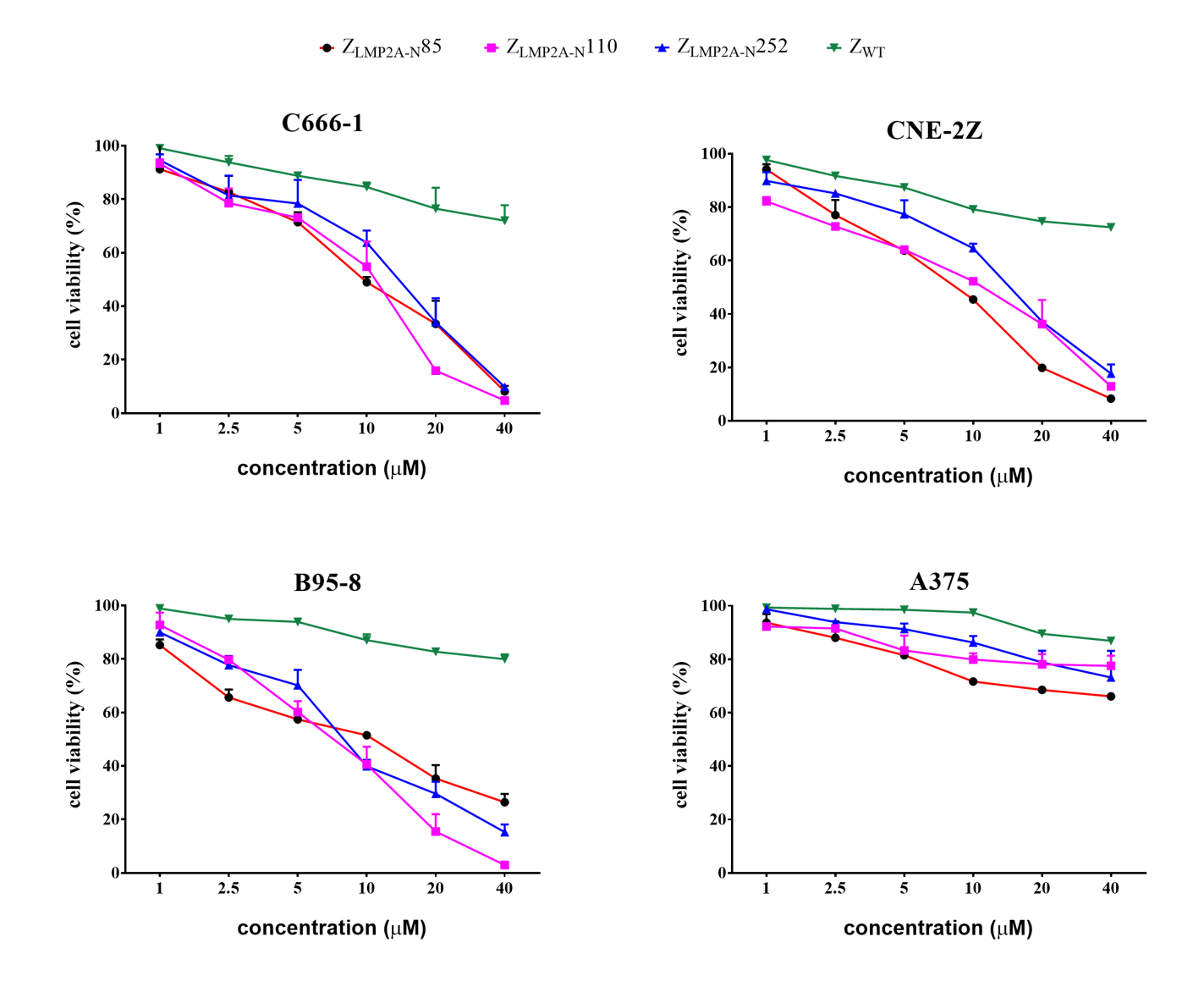

Supplement: Supplementary file 5 — Figure S4 [file 41419_2020_2410_MOESM5_ESM.tif]

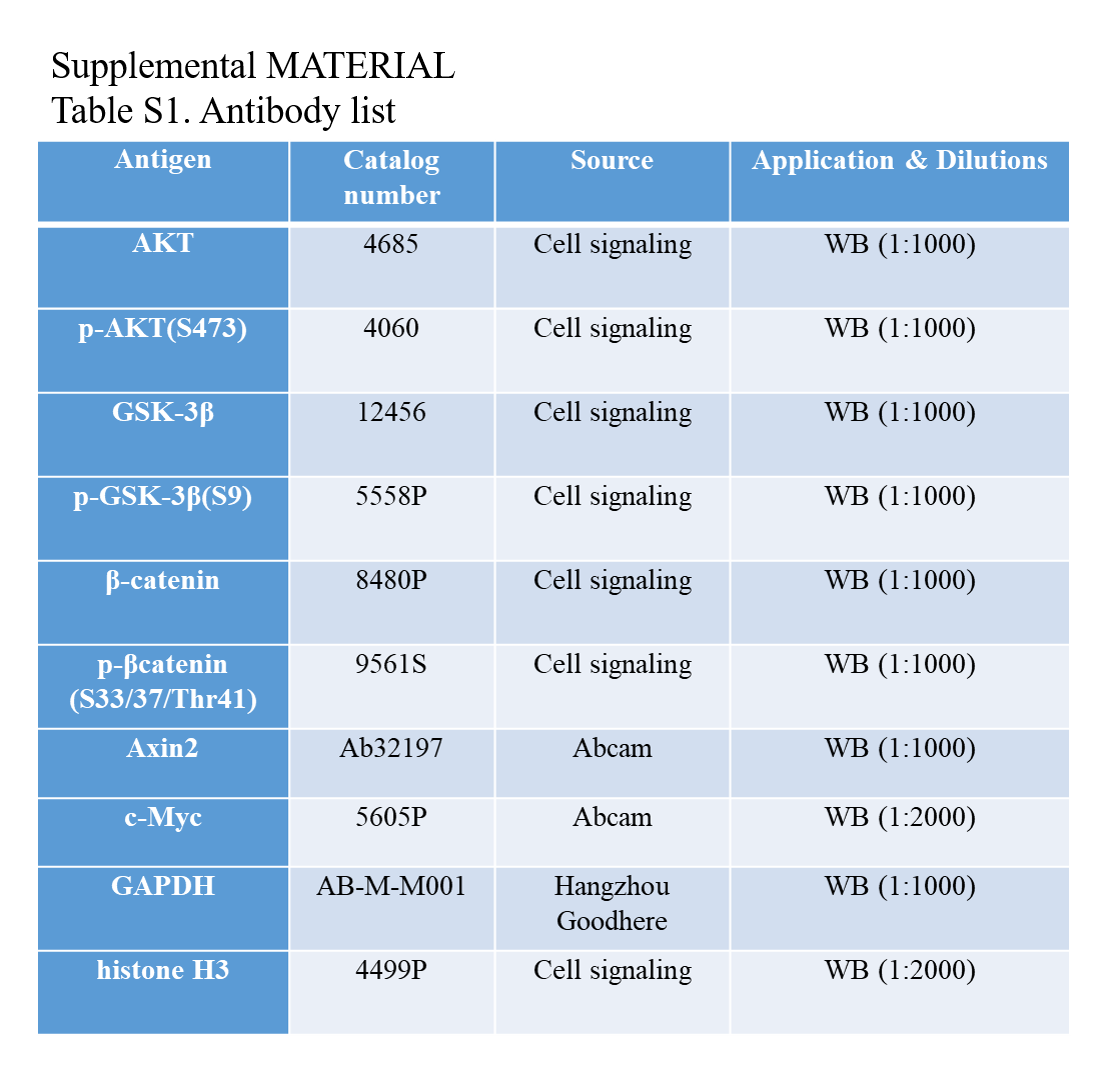

Supplement: Supplementary file 6 — Figure S-T1 [file 41419_2020_2410_MOESM6_ESM.tif]
